# Safety and efficacy of normobaric oxygenation on rescuing acute intracerebral hemorrhage-mediated brain damage—a protocol of randomized controlled trial

**DOI:** 10.1186/s13063-021-05048-4

**Published:** 2021-01-26

**Authors:** Zhiying Chen, Jiayue Ding, Xiaoqin Wu, Bing Bao, Xianming Cao, Xiangbin Wu, Xiaoping Yin, Ran Meng

**Affiliations:** 1 Department of Neurology, Xuanwu Hospital, Capital Medical University, Beijing, 100053 China; 2 Center of Stroke, Beijing Institute for Brain Disorders, Beijing, China; 3 Department of Neurology, Affiliated Hospital of Jiujiang University, Jiujiang, 332000 Jiangxi China

**Keywords:** Intracerebral hemorrhage, Normobaric hyperoxia, Cerebral perfusion, Randomized controlled trial

## Abstract

**Background:**

All of the existing medication and surgical therapies currently cannot completely inhibit intracerebral hemorrhage (ICH)-mediated brain damage, resulting in disability in different degrees in the involved patients. Normobaric oxygenation (NBO) was reported attenuating ischemic brain injury. Herein, we aimed to explore the safety and efficacy of NBO on rescuing the damaged brain tissues secondary to acute ICH, especially those in the perihematoma area being threatened by ischemia and hypoxia.

**Methods:**

A total of 150 patients confirmed as acute spontaneous ICH by computed tomography (CT) within 6 h after symptoms onset, will enroll in this study after signing the informed consent, and enter into the NBO group or control group randomly according to a random number. In the NBO group, patients will inhale high-flow oxygen (8 L/min, 1 h each time for 6 cycles daily) and intake low-flow oxygen (2 L/min) in intermittent periods by mask for a total of 7 days. While in the control group, patients will breathe in only low-flow oxygen (2 L/min) by mask for 7 consecutive days. Computed tomography and perfusion (CT/CTP) will be used to evaluate cerebral perfusion status and brain edema. CT and CTP maps in the two groups at baseline and day 7 and 14 after NBO or low-flow oxygen control will be compared. The primary endpoint is mRS at both Day14 post-ICH and the end of the 3rd month follow-up. The secondary endpoints include NIHSS and plasma biomarkers at baseline and Day-1, 7, and 14 after treatment, as well as the NIHSS at the end of the 3rd month post-ICH and the incidence of bleeding recurrence and the mortalities within 3 months post-ICH.

**Discussion:**

This study will provide preliminary clinical evidence about the safety and efficacy of NBO on correcting acute ICH and explore some mechanisms accordingly, to offer reference for larger clinical trials in the future.

**Trial registration:**

ClinicalTrials.gov NCT04144868. Retrospectively registered on October 29, 2019.

**Supplementary Information:**

The online version contains supplementary material available at 10.1186/s13063-021-05048-4.

## Background

Although the incidence of spontaneous intracranial hemorrhage (ICH) was reported as 10–30/100,000 worldwide, which only accounted for 10–15% in all stroke subtypes, both the burden of disability and the ratio of mortality were very higher than other stroke subtypes, even though they underwent medication and surgical treatment, resulting in poor clinical outcomes [1]. Whereby, new therapy for improving the outcome of ICH is urgently to be explored. Brain edema in the perihematoma area was reported to contribute a lot on ICH-mediated brain damage and closely associated with poor clinical outcomes, such as deterioration or even death [2–4]. Brain edema destroys the blood-brain barrier (BBB), resulting in inflammatory factors releasing into the blood circulation, further leading to the dysfunction of cerebral blood flow (CBF) automatic regulation, and finally, reducing the CBF in the perihematoma area [5]. Therefore, CBF improvement and brain tissue protection in the perihematoma area may be important targets in ICH treatment. However, no proper method is accepted on this aspect up to now.

Normobaric oxygenation (NBO) is a novel approach to elevate the pressure of oxygen in the ischemic penumbra [6], it can also exert neuro-protective effects against chronic ischemia and other hypoxic diseases, and moreover, it is considered as safe and convenient, especially in ICH setting clinically; unlike hyperbaric oxygenation (HBO), NBO required no special device and has no contraindication as HBO does, and moreover, NBO can be performed in bedside with lower cost [7]. The previous study revealed that early NBO could attenuate BBB damage and improve the outcome of ischemic stroke in patients with delayed rtPA treatment [8]. Research by Ding et al. demonstrated that NBO used in acute stage of ischemic stroke could inhibit lactic acidosis of the ischemic rat brain [9]. Other experimental studies revealed that the mechanisms of NBO on brain protection mainly included improving brain metabolism and increasing the tolerance of the brain to ischemic damage [10, 11].

An animal study assessing NBO with 90% concentration of oxygen 6 h daily for 3 consecutive days resulted in remarkable neuroprotection in rat ICH, presented as neurological function improvement, brain edema attenuation, downregulation of HIF-1a and VEGF expression, and apoptotic cells in the hematoma area diminished, when compared with control [12]. Some experimental studies showed that NBO reduced cerebral edema and played a neuroprotective role by stabilizing the integrity of BBB and improving perihematoma oxygen metabolism inhibiting the expression of HIF-1a, MMP-9, and Caspase-3 as well as increasing the expression of VEGF, ROS after ICH [12–16]. Whereby, we speculate that NBO may attenuate the ICH-mediated ischemic-hypoxic damage of the brain in the perihematoma area by improving cellular oxygenation and metabolism as well as protecting BBB in human.

To explore the safety and efficacy of NBO on rescuing acute ICH-mediated brain damage in a clinical setting, it is a crucial and indispensable step to conduct this clinical trial for the first time ahead of clinical transformation, based on previous exciting animal experiments results.

### Objectives

This protocol aimed to investigate the safety and efficacy of NBO on treating patients with acute ICH.

### Hypotheses

1. NBO does not increase the risk of hematoma enlargement and re-bleeding and even death.
2. NBO can improve cerebral perfusion in the perihematoma area and attenuate brain edema.
3. NBO will mitigate ICH-induced long-term neurological deficits.
4. NBO will protect the integrity of BBB.

## Methods and design

### Study design

**Fig. 1:**
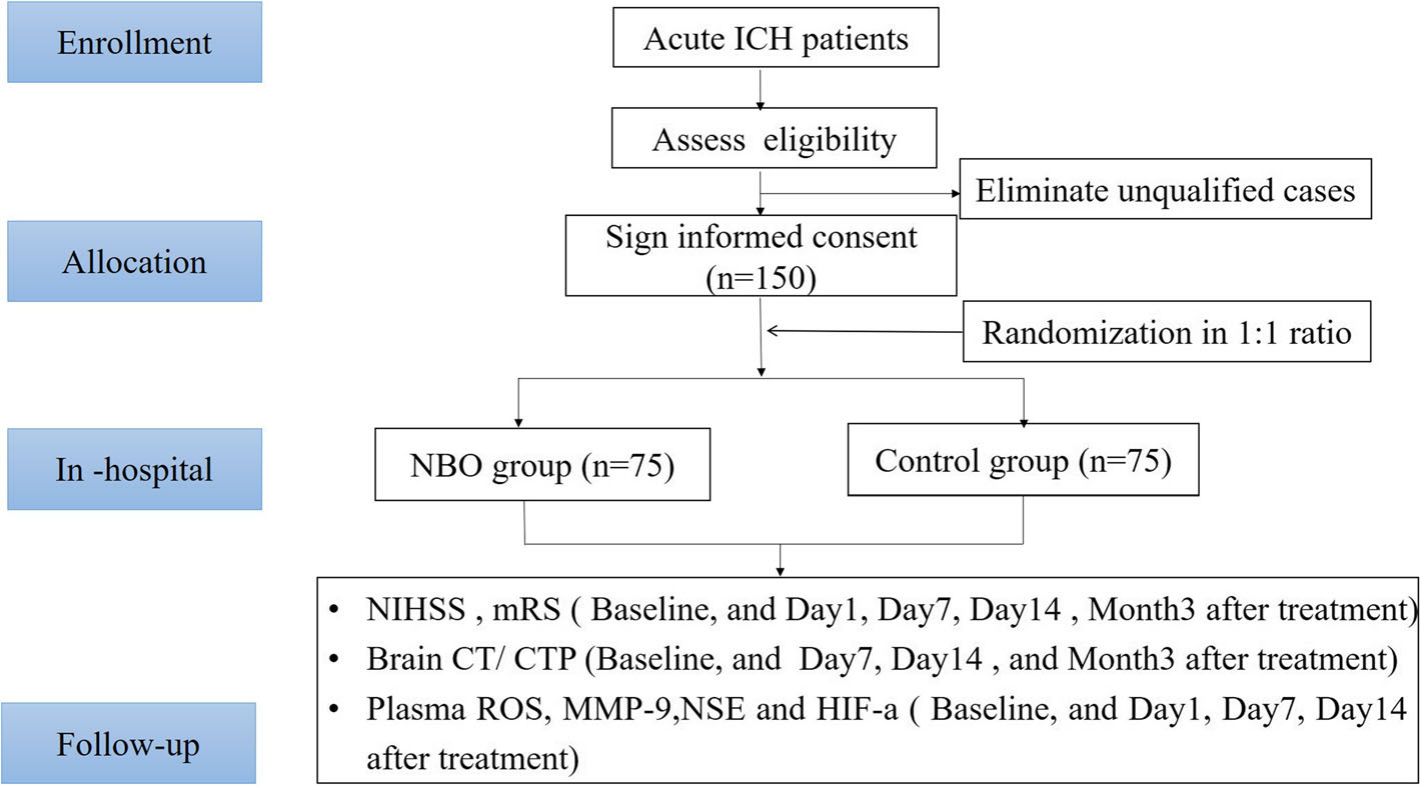
Flowchart of this study. NBO, normobaric oxygenation

This is a multi-center, randomized and controlled two-arm (1:1 ratio) superiority trial. A total of 150 patients will be immediately and randomly enrolled into NBO and control groups and undergo NBO or control treatment, respectively, after confirmed the diagnosis of acute spontaneous ICH by CT imaging and signed the informed consent. The flowchart of the study is displayed in Fig. 1.

### Eligibility criteria

Inclusion criteria include the following: (1) 18–80 years, (2) National Institute of Health Stroke Scale (NIHSS) ≥ 6 and Glasgow Coma Scale (GCS) > 8, and (3) signed informed consent.

Exclusion criteria include the following: (1) The history of previous ICH, ischemic stroke, and other cerebral injuries; (2) pre-ICH modified Rankin scale (mRS) ≥ 1; (3) Severe lung or heart diseases (forced expiratory vital capacity is less than 1.0 L or oxygen-dependent); (4) Austere and dying status; (5) history of brain surgeries; and (6) severe compliance.

### Study setting

This study was approved by the ethics committee of Xuanwu Hospital, Capital Medical University, and the Affiliated Hospital of Jiujiang University. When eligible patients are enrolled, their information will be sent to Dr. Chen, who will generate the allocation sequence and assign participants to interventions. After they understood the purpose and procedure of the study and their legal rights, they signed the informed consent by themselves or by their legally authorized delegates. On the informed consent form, the participant will be asked if he or she agrees to use their data. There is no anticipated harm and compensation for trial participation.

### Assignment of interventions: allocation

Sealed envelopes containing the allocation protocols (NBO or control) were labeled with sequential numbers prepared by a researcher who was not involved in the delivery of the interventions or the screening of subjects. Allocation was performed using a permuted block randomization procedure to ensure the 1:1 ratio of the patients in the two arms. The allocation sequence was determined using a computerized random number generator with block sizes of two subjects. The investigators will enroll the potential patients with a sequential number of opaque sealed envelopes, which would be opened only after obtaining the informed consent.

### Intervention

All ICH patients will undergo routine medication treatment. Moreover, patients in the NBO group will intervene with high-flow mask oxygen (8 L/min/1 h, 6 times daily, and 2 L /min in NBO intermittent periods), and the nurse on duty performed oxygen flow adjustment. Patients in the sham group were treated only with low-flow mask oxygen (2 L / min/ daily). At the same time, the nursing staff on duty supervises the implementation of oxygen flow and laboratory tests. Implementing NBO will not require alteration to usual care pathways (including use of any medication), and these will continue for both trial arms. If the study protocol needs to be changed, the sponsor is notified first, then PI notifies each center, and a copy of the modified protocol is sent to PI for adding to the investigator’s field file. Any violations are fully documented using the violation report form. We can also update protocols in clinical trial registrations.

NBO and control will be performed in the hospital under the supervision of an investigator. All patients and doctors were blinded to the allocation assignments. NBO inhalation can be ceased at any time if the patient reports any discomfort. The data monitoring committee (DMC) consists of principals, data managers, data monitors, and statistical analysts. It is independent from the sponsor and competing interests.

### Outcome measurement

#### Primary outcome

The mRS score is used as the primary outcome to evaluate the effectiveness of NBO. The proportions of the patients in the two arms with mRS ≥ 3 will be compared at both day 14 post-ICH and the end of the 3rd month follow-up.

#### Secondary outcomes

##### Plasma biomarker

In this study, blood samples will be collected from the peripheral veins of the patients for laboratory testing. Plasma biomarkers, which can represent brain and BBB injury (MMP-9, NSE) and oxidation (ROS, HIF-a), will be tested at baseline and days 1, 3, 7, and 14 after treatment. All blood samples will be centrifuged immediately after collection and stored in − 80 °C refrigerator until batch inspection.

##### Imaging assessment

**Fig. 2:**
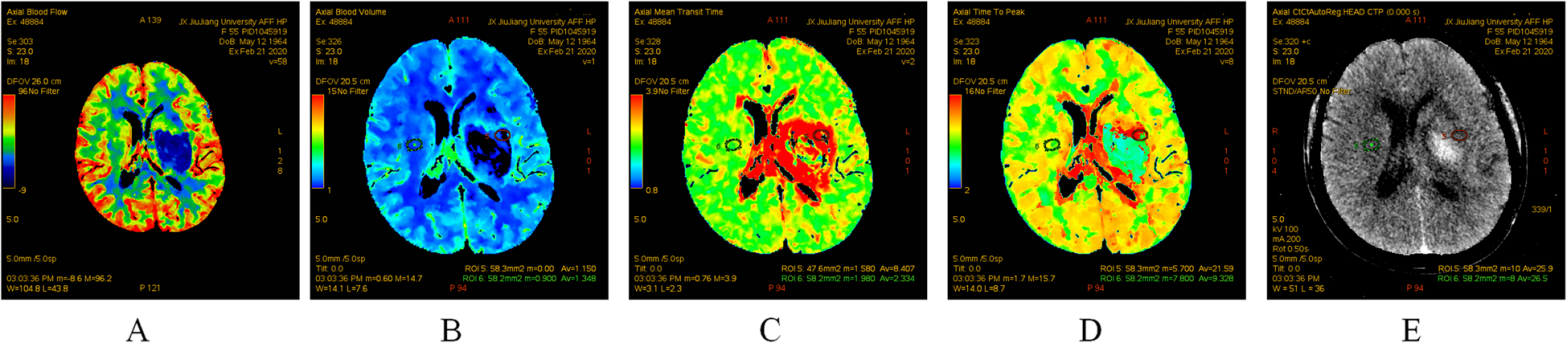
Maps of CT and CTP in an ICH patient example. **a** Cerebral blood flow (CBF). **b** Cerebral blood volume (CBV). b Mean transition time (MTT). **d** Time to peak (TTP). **e** CT brain scanning

After confirming the diagnosis of acute ICH by CT scan (GE 256-row ultra-high-end spiral CT, USA), patients will continue to undergo CTA and CTP scanning within 1 h to obtain baseline data. CTA maps will be used to assess the presence of vascular malformations such as aneurysms and arteriovenous malformations [5]. CTP map will be used to evaluate cerebral perfusion, including the parameters of cerebral blood flow (CBF), mean transition time (MTT), time to peak (TTP), and brain CT (Fig. 2). The CT and CTP will be followed up at days 7 and 14 after the treatment to assess the volumes of the hematoma and the surrounding edema as well as cerebral perfusion. Two independent evaluators who are blinded to the study protocol will analyze the data of CT and CTP.

##### Neurological function assay

NIHSS is considered as a standardized assessment tool on evaluating neurological function in the acute phase of stroke, which includes 15 items in 11 fields of different neurological status [17]. The average NIHSS scores in the two arms at every time point of baseline and days 1, 3, 7, and 14 and the end of the 3rd month after treatment will be assessed.

The mRS is the most comprehensive neurological disability measurement tool and is widely used in almost all recent acute stroke trials to evaluate the outcomes [18, 19], which assigns 7 disability levels, ranges from 0 (no symptom) to 5 (severe disability) and 6 (death) [20, 21]. The mRS in two arms will be assessed at baseline and the end of the end of the 3rd month after treatment, respectively.

The qualified investigators blinded to the protocol will be responsible for the NIHSS and mRS evaluation.

### Data management

All medical data required according to the protocol will be collected via the case report forms based on the hospital database, facilitating the real-time, central assessment of the data completeness and follow-up. As the questionnaire forms are received, a study researcher will make an inspection of the responses and inquire missing data when possible. The main analysis will be done using the available (not the imputed) data. For participants who consent to participate the trial but deviate from intervention protocols, i.e., refuse the randomized allocation, the follow-up data will still be collected the same as participants following the protocol, which will be analyzed according to intention to treat. The corresponding code table and informed consent form will be kept strictly in the file library. Medical records of the participants will be kept at the hospital. Researchers, research institutions, and ethics committees will be allowed access to the records. The study will not reveal the individual identities of the participants. Participants can request access to their personal information at any time and can modify the information if necessary.

### Clinical report form

An independent research physician will not be involved in the treatment and monitoring of patients in the inpatient ward and enter all necessary data into the prepared clinical report form (CRF). The CRF will be completed as soon as possible (Table 1). All missing data will be reasonably explained. The completed CRF page will be examined for completeness and reasonableness by the principal investigator and responsible supervisors.

**Table 1:**
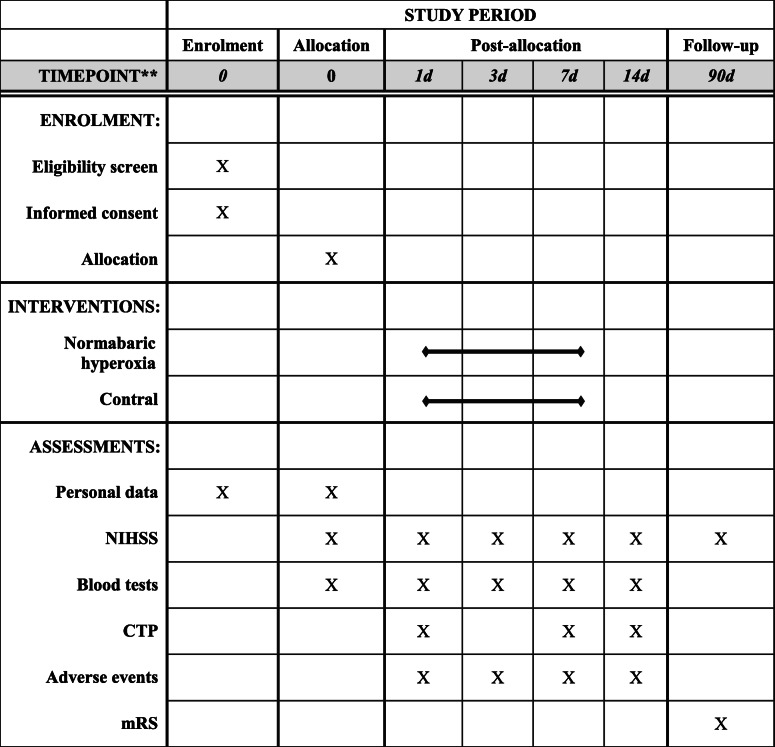
Flow chart of the trial

*CTP* computed tomography perfusion, *NIHSS* National Institute of Health stroke scale, *mRS* modified Rankin scale

### Sample size

According to previous NBO researches, GPower software will be used to calculate the case number and set the effective size to 0.25, α error probability to 0.05, power (1-β error probability) to 0.8, and the loss rate to 10%, the final result is that the total sample size is 150. Whereby, the goal in this study is to recruit 75 ICH patients in each arm.

### Recruitment

Two large stroke centers will participate in this clinical trial; both of them have sufficient clinical research experiences. We believe enough data of the ICH patients will be collected within the time protocol.

### Statistical analysis

For binary data, risk ratio and 95% of the confidence interval will be calculated. For continuous data, multivariate repeated measures or one-way analysis of variance analysis (assuming data are normally distributed) will be applied. We will regard the patients lost to follow-up as no event happening; even though there may be potential clinical events (such as ischemic stroke, recurrent stroke, and death) occurring in the future. All data will be analyzed on SPSS 20.0 (IBM Inc., Chicago, IL, USA) with the significance level of *P* < 0.05 (two sides). Missing value will be replaced using multiple imputations.

In addition to the analysis of primary and secondary outcomes, several subgroup analyses for distinct and relevant patient cohorts will be performed, which will involve age, sex, education, comorbidity, and hemoglobin.

### Interim analyses

Statistical analysis will be performed when the total number of samples collected reaches to 76 cases. The primary investigator will obtain these interim results and will decide whether to continue the experiment. We will stop the trial if the safety of the patients in the NBO group is much lower than that in the sham group in the outcomes of the interim data.

### Dissemination plans

We will publish the collected and statistical data in professional journals.

## Discussion

Brain injury in the perihematoma area was considered as one of the major causes related to poor outcomes of ICH patients [22]. Research also revealed that ICH-mediated perihematoma brain injury included cerebral edema, ischemia, and inflammation [22–26]. Unfortunately, no effective therapy can correct them at present.

Hypo-perfusion and hypoxia in the perihematoma area are the major mechanisms of ICH-mediated brain tissue injury, which may result from cerebral parenchyma microcirculation insufficiency caused by the compressing of hematoma or the toxicity of the bleeding released metabolites [5, 26]. Whereby, to rescue the perihematoma area by correcting hypo-perfusion, hypoxia may be the key step to obtain a good outcome.

Previous studies showed that NBO could rapidly increase the oxygen concentration in brain tissues and improve mitochondrial function [15, 16, 27, 28]. It could improve the oxygen concentration in the ischemic penumbra, protect mitochondrial function, and keep the BBB integrated in patients with acute cerebral infarction [15, 29, 30]. In patients with acute brain trauma, NBO increased brain metabolic rate, attenuated cerebral edema, reduced local brain cell acidosis, and protected the BBB [31].

The oxygen flow and frequency of NBO are commonly being concerned. Based on our previous NBO study in patients with cerebral venous disease [32], as more than 2 h continuous NBO may make the patients’ face at risk of respiratory alkalosis, whereby, the oxygen flow in this study is 8 L/min for 1 h, and the interval of two times of NBO is 4 h (a total of 6 times daily). Patients in the NBO group will undergo 8 L/min of oxygen immediately as soon as their acute ICH is confirmed.

To our knowledge, this is the first study about NBO on treating acute ICH. We will evaluate the cerebral blood flow and perfusion status in the perihematoma area dynamically (Fig. 2); moreover, we will explore probable mechanisms of NBO on brain protection in patients with ICH by testing plasma biomarkers dynamically.

The limitation of this study may be that this is only a two-center clinical study and the case numbers are not large enough to represent all ICH patients. Further multi-center study with a large number of patients is still needed.

### Trial status

The study is currently recruiting and enrolling participants according to version 2 of the protocol in October 2019. Recruitment began on Januaray 12, 2020, and the approximate date for completion of recruitment will be August 1, 2021.

The protocol is available from the corresponding author.

## Supplementary Information

**Additional file 1.**

## Data Availability

Data used or analyzed in this study may be provided upon reasonable request of the corresponding author.

## Abbreviations

NBO: Normobaric oxygenation
ICH: Intracerebral hemorrhage
CTP: Computed tomography perfusion
BBB: Blood-brain barrier
FiO_2_: Fraction of inspiration O_2_
MMP-9: Matrix metalloproteinase-9
HIF-1a: Hypoxia-inducible factor-1a
VEGF: Vascular endothelial growth factor
CO_2_: Carbon dioxide
CBF: Cerebral blood flow
NSE: Neuron- specific enolase
