# Supplementary material for: Safety and efficacy of normobaric oxygenation on rescuing acute intracerebral hemorrhage-mediated brain damage—a protocol of randomized controlled trial
Source: Trials. 2021 Jan 26;22:93. doi: 10.1186/s13063-021-05048-4 (PMC7836205; doi:10.1186/s13063-021-05048-4)
Supplement: Supplementary file 1 — Additional file 1. [file 13063_2021_5048_MOESM1_ESM.docx]

| Item Number | Reason |
| --- | --- |
| 11c | Participants in our intervention method have good compliance and there is no need to monitor compliance measures. |
| 11d | NBHO therapy does not conflict with other nursing measures. No special instructions are required. |
| 18b | The subjects had no side effects on NBHO intervention, and they were able to complete the trial and follow-up. |
| 20b | Our statistics include primary and secondary results without any additional analysis methods. |
| 25 | Participants in this trial have good compliance, and there is no need to spare or modify the research protocol. |
| 30 | The subjects had no side effects on NBHO intervention, which requires no compensation plan. |

This table is an explanation of the relevant items not written in the method.
